# Microalgae and co-culture for polishing pollutants of anaerobically treated agro-processing industry wastewater: the case of slaughterhouse

**DOI:** 10.1186/s40643-023-00699-4

**Published:** 2023-11-15

**Authors:** Dejene Tsegaye Bedane, Seyoum Leta Asfaw

**Affiliations:** Center for Environmental Science, College of Natural and Computational Sciences, Addis Ababa University, P.O. Box 1176, Addis Ababa, Ethiopia

**Keywords:** Biomass, microalgae, Nutrient removal-efficiency, Photobioreactor, Resource-recovery, Wastewater

## Abstract

Anaerobically treated slaughterhouse effluent is rich in nutrients, organic matter, and cause eutrophication if discharged to the environment without proper further treatment. Moreover, phosphorus and nitrogen in agro-processing industry wastewaters are mainly removed in the tertiary treatment phase. The objective of this study is to evaluate the pollutant removal efficiency of *Chlorella* and *Scenedesmus* species as well as their co-culture treating two-phase anaerobic digester effluent through microalgae biomass production. The dimensions of the rectangular photobioreactor used to conduct the experiment are 15 cm in height, 20 cm in width, and 30 cm in length. Removal efficiencies between 86.74–93.11%, 96.74–97.47%, 91.49–92.91%, 97.94–99.46%, 89.22–94.28%, and 91.08–95.31% were attained for chemical oxygen demand, total nitrogen, nitrate, ammonium, total phosphorous, and orthophosphate by *Chlorella* species, *Scenedesmus* species, and their co-culture, respectively. The average biomass productivity and biomass yield of *Chlorella* species, *Scenedesmus* species, and their co-culture were 1.4 ± 0.1, 1.17 ± 0.12, 1.5 ± 0.13 g/L, and 0.18, 0.21, and 0.23 g/L*day, respectively. The final effluent quality in terms of chemical oxygen demand, total nitrogen, and total phosphorous attained by *Chlorella* species and the co-culture were below the permissible discharge limit for slaughterhouse effluent standards in the country (Ethiopia). The results of the study showed that the use of microalgae as well as their co-culture for polishing the nutrients and residual organic matter in the anaerobically treated agro-processing industry effluent offers a promising result for wastewater remediation and biomass production. In general, *Chlorella* and *Scenedesmus* species microalgae and their co-culture can be applied as an alternative for nutrient removal from anaerobically treated slaughterhouse wastewater as well as biomass production that can be used for bioenergy.

**Graphical Abstract:**

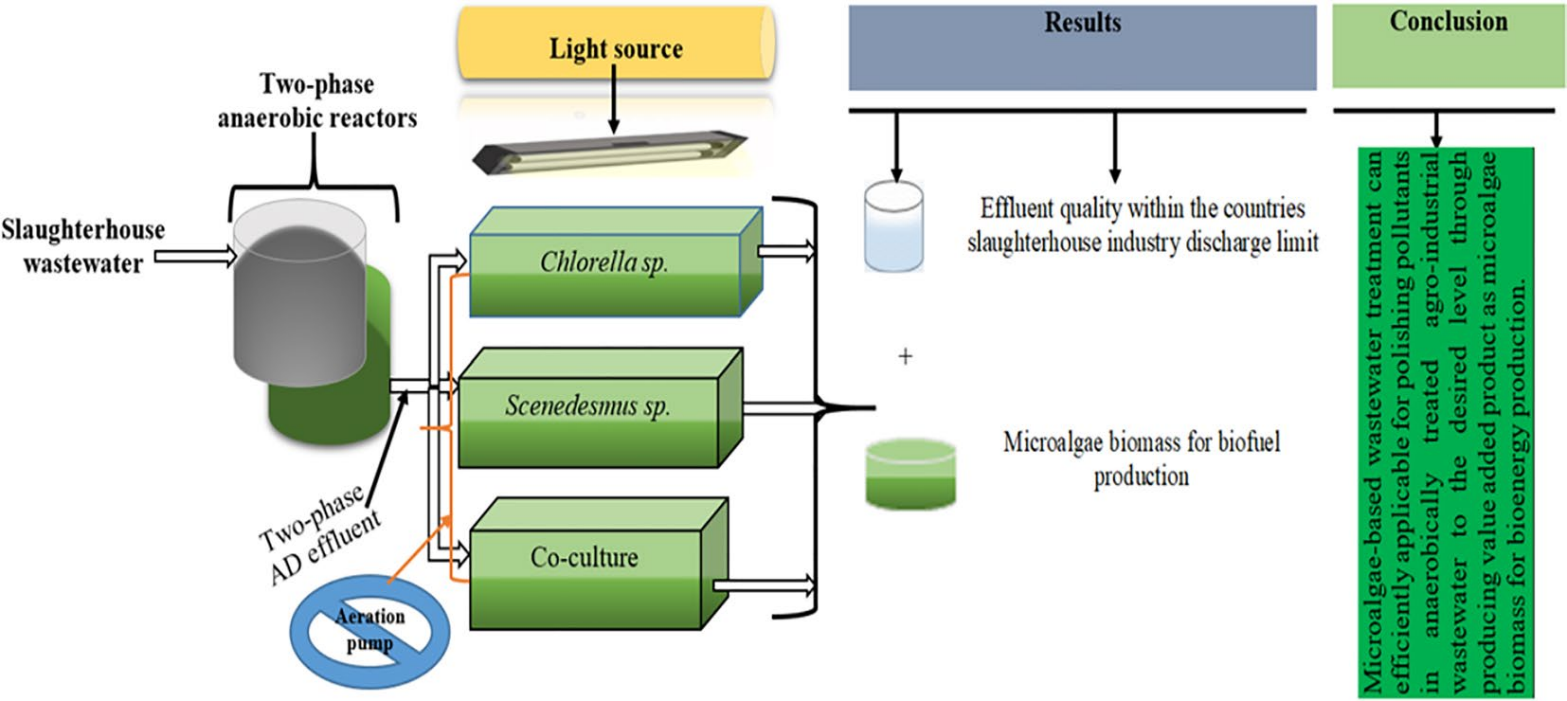

## Highlights

- Anaerobic reactor effluent supported microalgae biomass production
- Microalgae potentially remove residual organic matter and nutrients to the required level
- Biomass of the microalgae can be used for high valued products production
- Integration of microalgae at polishing step demonstrate an exercise of circular bioeconomy

## Introduction

In developing countries, more than ninety percent of the agro-processing industries discharge partially treated or untreated effluents recklessly into the environment. The effluents from the agro-processing industries as well as anaerobic digestion (AD) effluents are known for their high organic matter, nutrients, and other pollutants that are unsafe to the receiving water bodies or environment (Zemene Worku and Seyom Leta 2017; Ashekuzzaman et al. 2019; Bustillo-Lecompte and Mehrvar 2015; Hailu et al. 2020; Yirgu et al. 2020; K. Praveen et al. 2018; Leta et al. 2003), directing post-treatment process requirement to minimize or remove organic matters as organic and inorganic forms of phosphorus and nitrogen of the effluents (De Nardi et al. 2011; Chevalier et al. 2000; Dawana and Kassa 2020; Tsegaye and Leta 2022). If directly released, it results in eutrophication of the receiving water bodies and other environmental risks associated with greenhouse gas emissions from ammonia volatilization, groundwater nitrogen contamination, or pollution, and also affects human health via the food chain (Akar and Tunali 2005; Carey and Migliaccio 2009; Fornarelli, Bahri, and Moheimani 2017). The continuous discharge causes nutrient accumulation in water bodies such as the sea, lake, and river, which in turn results in rapidly growing nutrients plants on the surface of the water (algal bloom). The algal bloom blocks sunlight from the waterbed plant life. Because of the dead algal blooms, oxygen is reduced in the water, and microorganisms, which use up any remaining oxygen within the water, break down the dead plant matter. Finally, all animal life dies due to the lack of oxygen in the water environment. Hence, the concern over deteriorating freshwater bodies quality has led to more tough regulations governing the quality of wastewater discharge from agro-processing industries (Figueroa-Torres et al. 2021; Cai et al. 2013; Arbib et al. 2014). Several types of conventional post-AD treatment based on both physico-chemical and biological approaches involving different combinations of aerobic and anoxic stages have been exploited for the treatment of the nutrients and organic matter in the anaerobically treated agro-processing industry effluent. Unfortunately, these techniques often do not allow regular nutrient recovery due to the high investment and operational costs the industry incurred (De la Varga et al. 2013; Ruiz-Martinez et al. 2012) and also produce a huge amount of sludge (Craggs et al. 2014). Recent studies have shown that different microalgae species are emerging not only as a cost-effective but also sustainable agro-processing industry wastewater treatment as they are capable of instantaneously removing total nitrogen (TN), total phosphorous (TP), and the chemical oxygen demand (COD) through mixotrophic assimilation, which is combined with microalgae phosphorus luxury uptake that results in high TN, TP, and COD removals at comparatively short hydraulic retention time (HRT) (Cai et al. 2013; Abdel-Raouf et al. 2012; Arbib et al. 2014; Salama et al. 2017). Moreover, studies have confirmed the potential of microalgae for TN, ammonium (NH_4_^+^–N), TP, and phosphate (PO_4_^−3^–P) removal from partially treated agro-processing industry wastewater by an anaerobic digester through biomass production. TN,TP, NH_4_^+^–N, and COD removal efficiency ranging between 74 and 92%, 74 and 100%, 96 and 99%, and 77% were reported in Sacristán de Alva et al. (2013), L. Zhu et al. (2014), and Gentili (2014), respectively, as well as less carbon dioxide (CO_2_) being released during the cultivation of microalgae biomass.

However, integration of two-phase AD with microalgae growing or cultivation using a photobioreactor for nutrient recovery and biomass production remains rare (Onay 2018), and most of the studies used synthetic wastewater or microalgae growing media such as Bold’s Basal Medium (BBM) in flasks (J. Miranda et al. 2012; Mamo and Mekonnen 2020). But little has been done using partially treated agro-processing industry effluent for nutrient and organic matter removal as well as bioethanol and biodiesel production using *Scenedesmus* sp. cultivated in anaerobic digester effluent (Yirgu et al. 2020) and *Chlorella vulgaris*, *Scenedesmus dimorphus,* and their co-culture for treating diluted municipal wastewater (Asmare et al. 2014). Furthermore, the microalgae-growing materials in most of the research were cylindrical or conical glass or flasks with a capacity of less than five liters (Yirgu et al. 2020; Asmare et al. 2014). The co-culturing of microalgae as photosynthetic organisms has been assumed to have both cooperative associations by exchanging metabolites, leading to the ultimate enrichment of biomass productivity and consequently increasing the nutrient removal efficiency, and competitive associations resulting in the secondary discharge of metabolites (known as allelochemicals) (Gururani et al. 2022; Goh et al. 2022; Bacellar Mendes and Vermelho 2013; Renuka et al. 2013; Gonçalves et al. 2017). Furthermore, these particular interactions among the microalgae in co-culture have numerous benefits for the treatment of agro-processing industry wastewater processes, including slaughterhouses, the promotion of the cell division process, the enhancement of the consumption or reduction of complete nutrients, the introduction of allelochemical production, the resistance to contaminants and predators, and the formation of a settleable system by the mixture of a single-cell organism with flocculating ones (Gururani et al. 2022; Renuka et al. 2013). Furthermore, the utilization of microalgae co-cultures or consortiums in wastewater treatment promises the achievability of the decontamination process as the loss of the first microalgae can be equilibrated by the second incorporated microalgae in the co-culture (Goh et al. 2022; Renuka et al. 2013). Though the application of Chlorella and Scenedesmus species for agro-processing industry wastewater remediation has been extensively reported with comparable or even better performance relative to this study, as far as our knowledge is concerned, the potential of coupling the two-phase anaerobic digestion system treating slaughterhouse wastewater with microalgae isolated from local freshwater bodies cultivated in a photobioreactor for COD, TN, NH_4_^+^–N, TP, and PO_4_^−3^–P removal as well as biomass production removal as well as biomass production is not studied so far. Therefore, the main objective of this study was to evaluate the COD, TN, NH_4_^+^–N, nitrate (NO_3_^−^–N), TP, and PO_4_^−3^–P removal efficiencies as well as the biomass production potential of *Chlorella* and *Scenedesmus* species microalgae, and their co-culture cultivated in photobioreactor treating two-phase anaerobically treated slaughterhouse effluent.

## Materials and methods

### Microalgae sample collection, isolation and cultivation

Freshwater sample from which *Chlorella* and *Scenedesmus* species microalgae isolated were collected from a local freshwater body, Awassa Lake, Awassa, Ethiopia, and transported to the laboratory. The collected sample was relocated to the closed flasks on arrival in the laboratory to avoid contamination, enriched in BBM, and then incubated using petridishes for 5 days at 25 °C with a light intensity of about 40–50 μmolm^−2^ s^−1^, as described in (Chalivendra 2014). The two microalgae species identification or isolation was done as indicated in Andersen and Kawachi (2005), Dolganyuk et al. (2020), K. Lee et al. (2014), and Ogbonna (2015) using agar plating with pipetting and serial dilution combinations based on their morphology using a light microscope. The isolated *Chlorella* and *Scenedesmus* microalgae species were again cultured in BBM until the required amount was obtained and stored in the refrigerator at 4 °C until used for the treatment of two-phase anaerobic digester effluent. Then 800 cm^3^ of isolated microalgae inoculums cultured in BBM and 7200 cm^3^ were fed to the photobioreactors. The components of the microalgae co-culture are *Chlorella* and *Scenedesmus* species at 1:1 monoculture inoculums on volume basis. The per liter BBM was composed of KH_2_PO_4_ (175 mg), K_2_HPO_4_ (75 mg), MgSO_4_ ·7H_2_O (75 mg), CaCl_2_ ·2H_2_O (25 mg), NaNO_3_ (250 mg), NaCl (25 mg), and H_3_BO_3_ (11.42 mg), 1 mL of microelement stock solution ZnSO_4_·7H_2_O (8.82 g), MnCl_2_ ·4H_2_O (1.44 g), MoO_3_ (0.71 g), CuSO4 ·5H_2_O (1.57 g), and Co(NO_3_)_2_·6H_2_O (0.49 g) in one liter), 1 mL of solution-1 of Na_2_EDTA (50 g) and KOH (3.1 g) in one liter, and 1 mL of FeSO_4_ (4.98 g and concentrated H_2_SO_4_ per liter) and pH 6.8. The solution was always autoclaved for 15 min at 121 °C before use.

### Inoculation and cultivation of the microalgae in AD effluent

#### Microalgae culturing conditions

The freshwater sample containing the microalgae was transferred and primarily grown in 250 mL Erlenmeyer flasks containing 100 mL of BBM medium at 22 ± 2 °C with cool white fluorescent lamps, giving a continuous light intencity of 40–50 μmolm^−2^ s^−1^. Air and CO_2_ were bubbled using an aeration pump at a flow rate of 250 mL/min and 100 mL/min, respectively, to produce adequate microalgae cultures for the wastewater treatment and biomass production experiments.

### Photobioreactor setup and operation condition

**Fig. 1:**
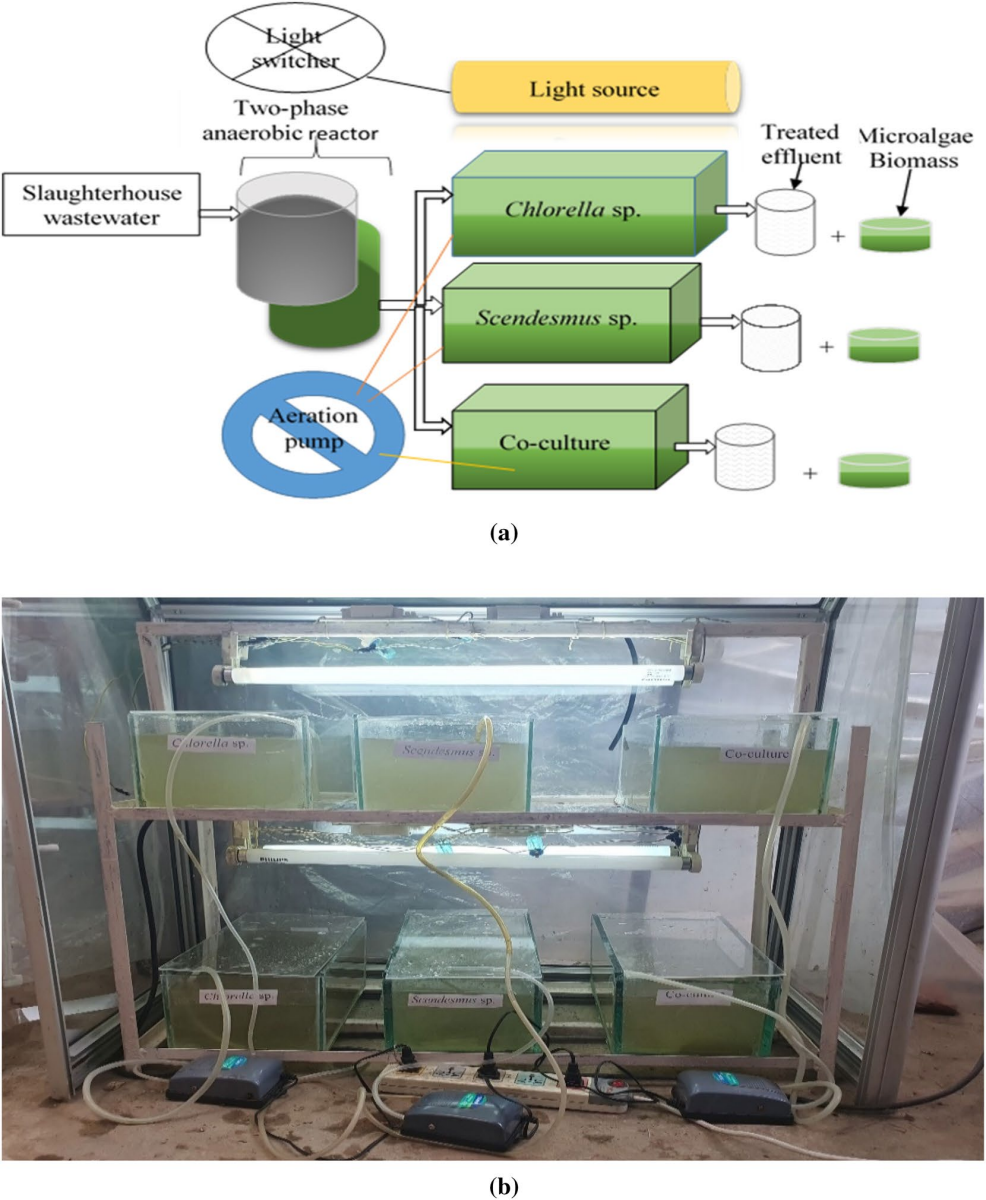
Photobioreactor setup for microalgae-based bioremediation experiment: **a** schematic and **b** photo

A rectangular photobioreactor was used to cultivate microalgae for the post-AD effluent treatment. The dimensions of the photobioreactor were 15 cm in height, 20 cm in width, and 30 cm in length. The total volume of the photobioreactor was 9000 cm^3^, with a working volume of 8000 cm^3^. Figure 1 shows a microalgae photobioreactor experimental setup; (a) schematic and (b) photo. In order to avoid contamination, the bioreactors were covered with transparent plastic glass. Two fluorescent lamps (20 watts each, Philips) with a maximum light intensity of 150–300 μmolm^−2^ s^−1^ above the surface of the photobioreactor was used as a light source. An electric timer switch controlled the photoperiod at a 12:12 light/dark cycle at room temperature. An aerator was used to supply air and CO_2_ at a flow rate of 250 and 100 mL/minute, respectively. The microalgae cultivation was run in duplicate for 20 days. The effluent of the two-phase AD system was fed to the photobioreactor in semi-continuous (draw and feed) mode. After 20-day incubation period the microalgae biomass in the photobioreactor drawn and another batch was fed. The effluent was filtered using 21-mm Whatman filter paper prior to feeding. After the twenty-day incubation period or end of the experiment, the microalgae’s biomass was harvested by evaporation. The effluent was then analyzed for physico-chemical parameters.

### Biomass, chemical oxygen demand, and nutrient removal analyses

#### Microalgae biomass and productivity

Microalgae biomass yield and productivity were determined according to (Lee et al. 2013) by measuring the optical density (OD) at 680 nm (OD680). The OD of microalgae was measured using JENWAY spectrophotometer.

$${\text{Microalgae biomass concentration }} = \, 0.{95} \times {\text{OD}}_{{{68}0}} - 0.0{4}$$

$$\mathrm{BP}=\frac{\mathrm{Ct}-\mathrm{Co}}{\mathrm{Tt}}$$

where Ct and Co represent the microalgae’s biomass at time (t) and initial time (t_0_), respectively.

#### Photobioreactor effluent quality analysis

The microalgae can utilize the main nutrients like carbon, phosphorus, and nitrogen that are required for growth from numerous wastewater sources, diluted and secondary to agro-processing industry effluent or municipal wastewater (Asmare et al. 2014; Yirgu et al. 2020; Passos et al. 2015; Cai et al. 2013). In this study, pollutant (COD, TN, NH_4_^+^–N, NO_3_^−^–N, TP, and PO_4_^−3^–P) removal efficiency evaluation of two microalgae, namely *Chlorella* sp. and *Scenedesmus* sp., isolated from freshwater of a local lake, and their co-culture were used to identify and select robust and suitable microalgae capable of treating slaughterhouse wastewater treated partially using a bench-scale two-phase AD system.

#### Chemical oxygen demand, and nutrient removal efficiency analyses

The temperature of the surface above the photobioreactor and the wastewater microalgae’s mixture pH were measured using a pH meter (Jenway, Manchester, UK). During the final water quality analysis, the photobioreactor effluent was centrifuged at 4500 rpm for about 10 min to separate the microalgae from the water, and then the supernatant was filtered. The concentrations of COD, TP, TN, NH_4_^+^–N, and NO_3_^−^–N were analyzed on 0, 4, 8, 12, 16, and 20 days of incubation time by taking a 50 mL sample according to the standard methods indicated by Dalrymple et al. (2013) using a Jenway spectrophotometer. Samples were filtered using Whatman GF/F filters before analysis. Nutrients or organic matter removal efficiencies were determined by:

$$\mathrm{Nutrient\, removal\, efficiency }\left(\mathrm{\%}\right)= \frac{\mathrm{Influent\, concentration}-\mathrm{effluent\, concentration}}{\mathrm{Intial\, concentration}}*100$$

The removal rate of the parameters (nutrients and organic matters) was calculated using the following equation.

$$\mathrm{Rr}=\frac{\mathrm{Ct}-\mathrm{Co}}{\mathrm{Tt}}$$

where Rr is COD, TP, TN, NH_4_^+^–N, and NO_3_^−^–N removal rate, Ct and Co represents the parameters concentration at time (t) and initial time (t_0_), respectively.

### Data analysis

The raw data collected during this experiment was entered into Microsoft Excel for further analysis. The result was presented in tables as the mean, standard deviation, and figures. Origin 22 statistical software was used to draw figures and perform descriptive analysis. The data obtained were analyzed to determine the degree of significance and for the comparisons of mean concentration or results of the two microalgae and their co-culture using one-way analysis of variance (ANOVA) using Minitab statistical software followed by a post hoc test at p ≤ 0.05.

## Results and discussions

### Feedstock characteristics

Two-phase AD effluent COD, TN, NH_4_^+^–N, NO_3_^−^–N, TP, and PO_4_^−3^–P concentrations subjected to treatment with *Chlorella* sp., *Scenedesmus* sp., and co-culture varied between 905–919, 359–376, 330–365, 89–102, 93–105, and 61–88 mg/L, respectively. Related study results showed that the COD, TN, NH_4_^+^–N, NO_3_^−^–N, TP, and PO_4_^−3^–P concentrations were varying between 97–1100 mg/L, 163–410 mg/L, 21–237 mg/L, 22–265 mg/L, 12–221 mg/L, and 0.6–170 mg/L, respectively, and can be used as a nutrient and carbon source for microalgae growth (Bakraoui et al. 2023), revealing the two-phas AD effluent supports microagae growth. Scholars also reported that the residual TN, TP, COD, and several micronutrients in the anaerobically treated agro-processing industry effluent can potentially support microalgae cultivation (Elvira E. Ziganshina et al. 2022; Bauer et al. 2021; L. Zhu, Yan, and Li 2016; Tambone et al. 2017).

### Operating environmental conditions during microalgae cultivation

#### Light intensity, temperature, and pH

The two microalgae species identification or isolation was done as indicated in Andersen and Kawachi (2005), Dolganyuk et al. (2020), K. Lee et al. (2014), and Ogbonna (2015) using agar plating with pipetting and serial dilution combinations based on their morphology using a light microscope.

For the photosynthetic organisms such as microalgae, the metabolic processes linked with nutrient assimilation for microalgal growth are determined by light. Microalgae species-specific light intensity needed for optimal growth was reported to be between 150 and 400 μmolem^−2^ s^−1^ for *Scenedesmus* species (Mostafa et al. 2012) and 200 to 500 μmolem^−2^ s^−1^ for *Chlorella* sp. (Maltsev et al. 2021), while optimum biomass production of both species of microalgae was at 150 μmolem^−2^ s^−1^ (Nzayisenga et al. 2020). Self-shading, increased transmittance pathways, and light attenuation can result in a reduction of biomass productivity at light intensity below a species threshold range while oxidative damage by photoinhibition occurs above the range (Whitton et al. 2015; Tan et al. 2022). Bench-scale photobioreactors overcome this by adjusting the light intensity in a limited range of 150–300 μmolem^−2^ s^−1^ (Gordon and Polle 2007; Singh and Singh 2015; Mostafa, Shalaby, and Mahmoud 2012). Therefore, the light intensity of the fluorescent lamp used for this study varied between 150 and 300 μmolem^−2^ s^−1^ which is in the range of previouse report.

The other factor that can affect microalgae nutrient removal efficiency and biomass production is temperature, and they are directly proportional to each other until the maximum threshold. In this study, the temperature of the surface above the reactor during the experimental period varied from 28.2 to 32.5 °C. A temperature range between 15 and 31.5 °C is assumed to be optimal for microalgae photobioreactors, with a critical maximum temperature that depends upon specific species, providing the nutrient concentration, and light supply not being limiting factors (Singh and Singh 2015; Andersen and Kawachi 2005).

Most microalgae have an optimum pH range for their photosynthesis and growth in between 7 and 11, but there are microalgae species that can grow in acid conditions as low as pH 1 (Whitton et al. 2015). In this study, the pH of the photobioreactor in which *Chlorella*, *Scenedesmus,* and co-culture were used to treat the two-phase AD effluent varied from 7.53 to 11, 7.31 to 10.6, and 6.7 to 11.5, respectively, which is consistent with the previous reported pH values for microalgae cultivation for pollutant reduction and biomass production (Bohutskyi et al. 2016; Asmare et al. 2014; Kusmayadi et al. 2022; Acevedo et al. 2017; Chevalier et al. 2000).

### Photobioreactor effluent quality

#### Nitrogen, nitrate and ammonium removal

**Fig. 2:**
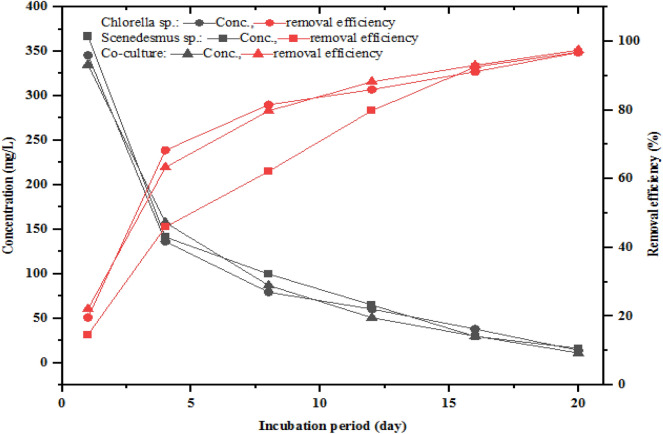
Variation of total nitrogen concentration and removal efficiency

The TN concentration of the two-phase AD effluent fed to the photobioreactor in which *Chlorella*, *Scenedesmus*, and the co-culture grown was 367.33 ± 8.50 mg/L. The photobioreactor effluent TN concentration treated by *Chlorella*, *Scenedesmus*, and co-culture varied between 10 and 17, 12 and 15, and 9 and 14 mg/L, respectively. The lowest TN concentration levels of 10, 12, and 9 mg/L were observed for the photobioreactor effluent in which *Chlorella*, *Scenedesmus*, and co-cultures were grown, respectively. The changes in TN concentration and removal efficiencies during the experimental period are shown in Fig. 2. In all three treatments, the concentration of TN decreased sharply in the 1^st^ eight days but steadily afterwards. The final concentrations of TN in all the treatments were below 15 mg/L. Furthermore, 60% TN removal efficiency was achieved on the 8^th^ day of the incubation or experimental period, and about 95% TN removal efficiency was achieved at the end of the 20 days by all three treatments. Besides, the TN removal rate or uptake by *Chlorella*, *Scenedesmus*, and co-culture was 20.80, 20.82, and 20.95 mg/L*day, respectively (Table 1).

**Table 1:** Removal rate of COD, TN, NO3-, NH4 + -N, TP, and PO4 − 3-P

Parameter	*Scenedesmus sp.*	*Chlorella sp.*	Co-culture
	Rr (mg/L*day)	Rr (mg/L*day)	Rr (mg/L*day)
COD	39.47	41.82	42.37
TN	20.82	20.8	20.95
NH_4_^+^-N	17.22	18.42	18.5
NO_3_^−^–N	4.3	4.35	4.37
TP	4.42	4.67	4.625
PO_4_^−3^–P	3.23	3.32	3.38

As stated in the different literature, the TN removal efficiency of AD effluent treated by microalgae between 90 and 100% depends on operating conditions, microalgae used, reactor type, and other factors (Yirgu et al. 2020). A study conducted by Shayesteh et al. (2021) using *Chlorella* species for agro-processing wastewater treatment indicated that the TN nutrient assimilation by microalgae was 75.50% with a final effluent concentration of 84.24 mg/L, while 76–95% of the TN (Cho et al. 2011; S. Zhu et al. 2022; Kim et al. 2013) during the biomass cultivation of *Chlorella* species and *Scenedesmus* species cultivation for biomass production using tannery wastewater removed 88% of the TN (da Fontoura et al. 2017). Similarly, M. K. Ji et al. (2013) studied the nutrient removal efficiency of *Chlorella* and *Scenedesmus* species and achieved an almost complete removal of TN by both species. Other studies also reported that the microalgae consortium had a TN removal efficiency of 67% (X. Hu et al. 2019a, b). The TN removal efficiency obtained in this study is consistent with the previous research findings for the partially treated agro-processing industry wastewater feedstock using *Chlorella* species and *Scenedesmus* species (Darpito et al. 2015; Farooq et al. 2013; Cai et al. 2013).

Microalgae uses nitrogen build up the cells’ components, like energy transfer molecules, enzymes, vitamins, genetic material, proteins, amides, hormones, and alkaloids. Furthermore, based on dry weight, it is the 2nd most abundant element next to carbon, making up 6–10% of microalgae biomass. The microalgae usually uptake the inorganic nitrogen forms nitrate, nitrite, and ammonium in the AD effluent (Yirgu et al. 2020; Passos et al. 2015). Microalgae assimilate the nitrogen nutrients that are found in wastewater in the preference of organic-N < NO_2_^−^ < NO_3_^−^ < NH_4_^+^–N via translocation across the cell membrane (Cai et al. 2013; Whitton et al. 2015), indicating NH_4_^+^-N is highly favored by microalgae (Cai et al. 2013; Yecong Li et al. 2011).

**Fig. 3:**
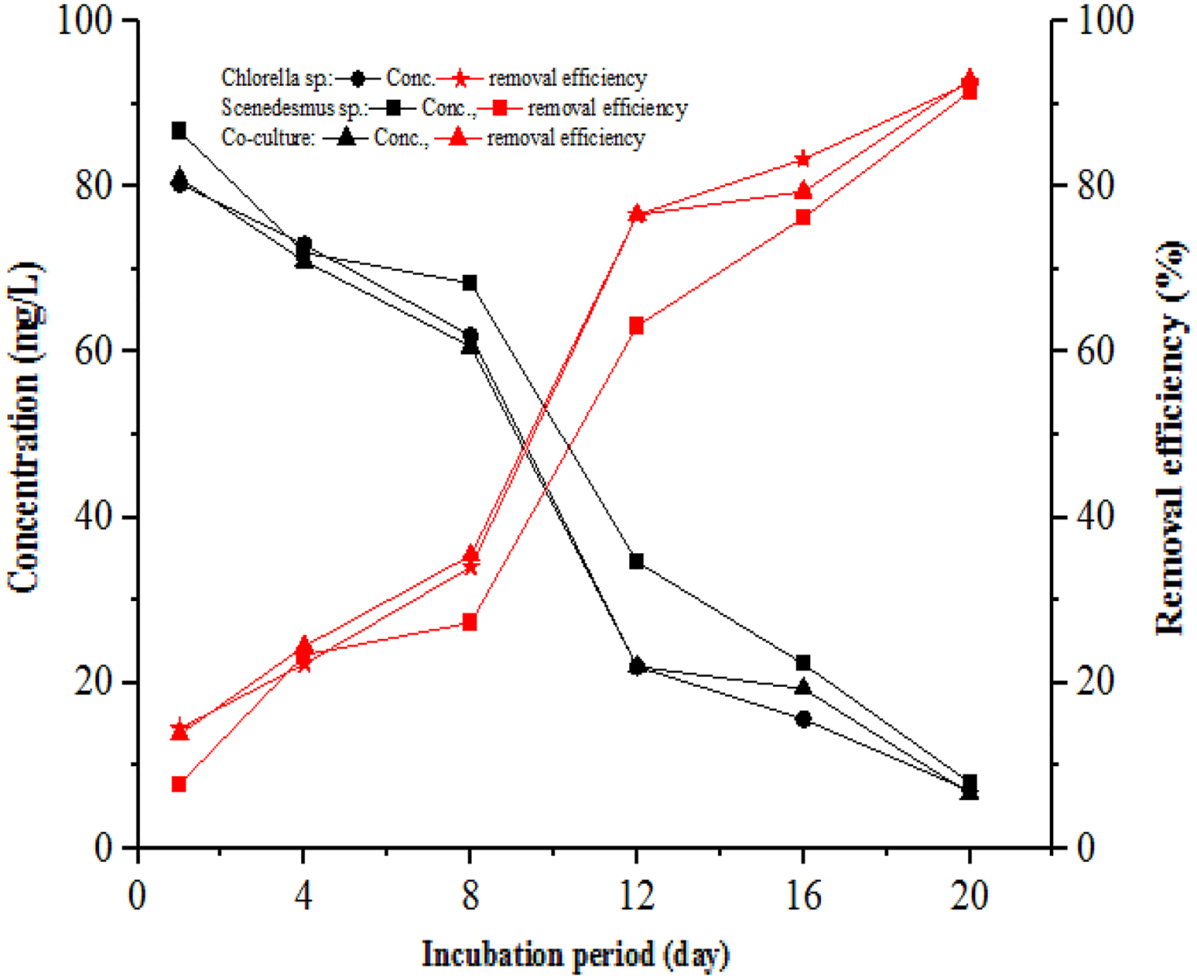
Variation of nitrate concentration and removal efficiency

The NO_3_^−^–N concentrations of two-phase AD effluent treated by *Chlorella*, *Scenedesmus*, and mixture were between 6 and 10, 6–8, and 5–8 mg/L, respectively. The NO_3_^−^–N concentrations in all treatments showed a steady and sharp decrease in the first 8 days and afterwards of treatment period, respectively (Fig. 3). Hence, low removal efficiency (RE) of NO_3_^−^–N was observed in the first 8 days of the incubation period. A NO_3_^−^–N RE of more than 60% was achieved after 12 days of the incubation or experimental period for all the microalgae and their mixture. Furthermore, the final effluent NO_3_^−^–N concentrations treated with *Chlorella*, *Scenedesmus*, and co-culture were below 10 mg/L with 91.08, 92.91, and 93.28% RE, respectively. The moderate decrease followed by a sharp increase in NO_3_^−^–N RE in the 1^st^ week of the incubation periods and afterwards, respectively, was attributed to the less preference of NO_3_^−^–N by microalgae when the NH_4_^+^–N concentration is enough to support the microalgae’s cell growth. The NO_3_^−^–N removal efficiency result achieved in this study is consistent with the previous study findings, as depicted below. Nitrate is the second inorganic nitrogen that microalgae prefer for growth, next to ammonium (Whitton et al. 2015). Studies have shown low (53%), 90%, and 100% NO_3_^−^–N removal efficiencies by *Chlorella* sp., from partially treated agro-processing industry wastewater and lecheate (D. Hu et al. 2021; Shi et al. 2007; Godos et al. 2009; Ajala and Alexander 2020).

**Fig. 4:**
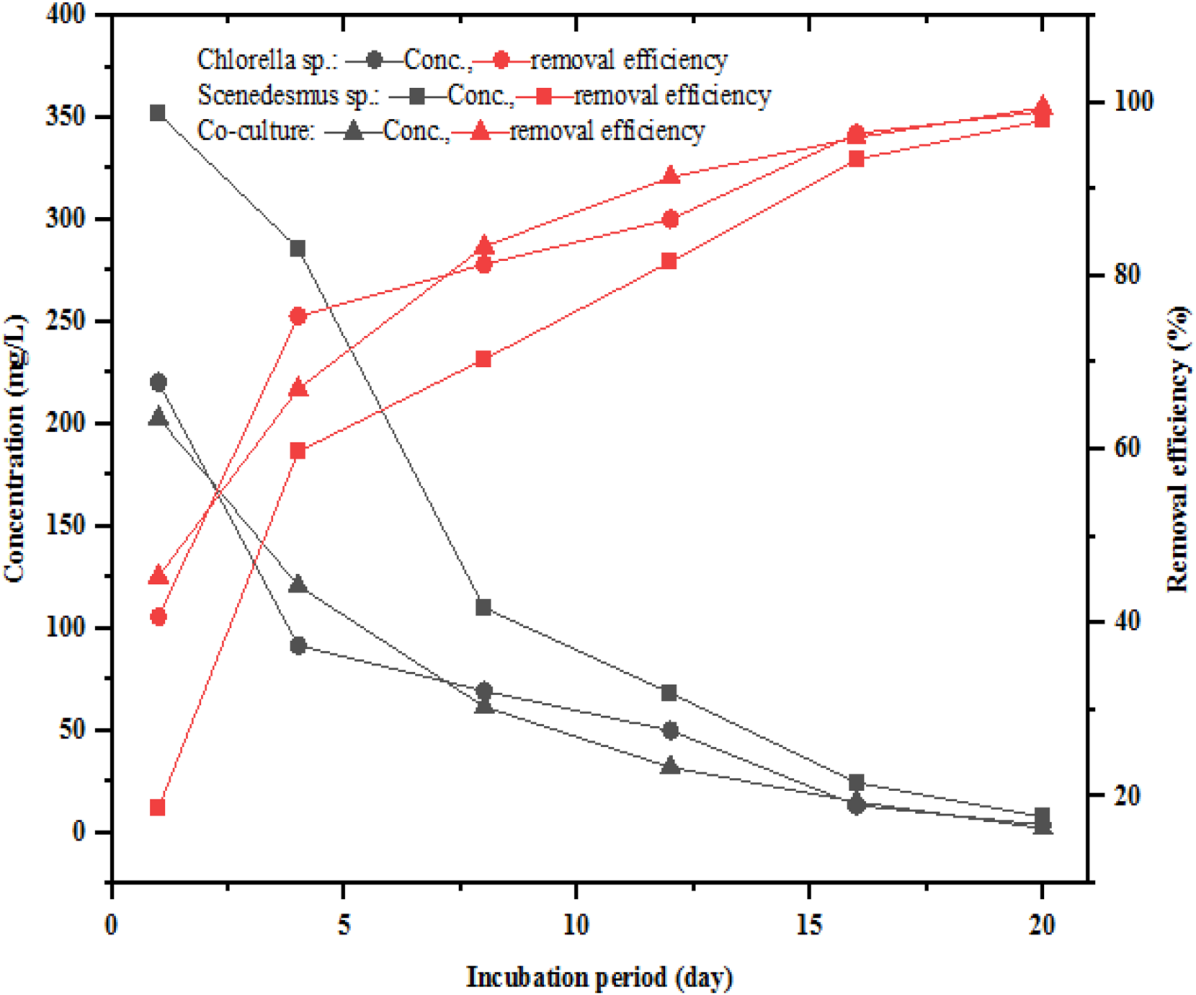
Variation of ammonium concentration and removal efficiency

Figure 4 shows the change in NH_4_^+^–N concentrations and removal efficiency during the experimental period by *Chlorella*, *Scenedesmus*, and co-culture. As indicated in Fig. 4, NH_4_^+^–N concentrations progressively decreased while the RE increased with time in all treatments. The decrease and increase of the concentration and RE of NH_4_^+^–N were high during the first 8 days of the incubation period and slow afterwards, respectively. Furthermore, the NH_4_^+^–N RE is higher than other nitrogen forms, which may be attributed to its preference for microalgae over other forms. Accordingly, in all three treatments, more than 50, 75, and 61% removal efficiency of NH_4_^+^–N were achieved in the 1, 8, and 12 days of th incubation or experimental period, respectively. The final NH_4_^+^–N concentrations were 3.67 ± 1.53, 7.67 ± 1.53, and 2.00 ± 1 mg/L, with removal efficiencies of 97.94, 99.01, and 99.46% for *Chlorella*, *Scenedesmus*, and co-culture, respectively. After 20 days of incubation, the lowest NH_4_^+^–N concentrations of 2, 6, and 1 mg/L were recorded in the photobioreactor with *Chlorella*, *Scenedesmus*, and co-culture, respectively. Simultaneously, after 20 days of incubation, the NH_4_^+^–N removal rates by *Chlorella*, *Scenedesmus*, and co-culture were 18.42, 17.22, and 18.50 mg/L*day, respectively (Table 1). The higher removal efficiency of NH_4_^+^-N was achieved by the co-culture of microalgae than monocultures, which is supported by earlier findings by Cai, Park, and Li (2013), though it was reported that *Chlorella* species can effectively tolerate NH_4_^+^–N. Similarly, (Scarponi et al. 2021) reported NH_4_^+^-N removal efficiency of 99.2% and 98.146% from organic waste digestate by *Chlorella and Scenedesmus* cultures, respectively, which is consistent with this study finding.

The higher removal efficiency and removal rates of NH_4_^+^-N than NO_3_^−^–N in all treatments were due to their consumption, preferably by microalgae for the growth of cells. Furthermore, the higher removal efficiency of NH_4_^+^–N is attributed to a reduction in culture pH, which in turn shifts the equilibrium from NH_3_ toward NH_4_^+^–N, which the microalgae prefer for their growth as a source of nutrients. Furthermore, this may be due to the lower energy requirement for NH_4_^+^–N assimilation of glutamine and the release of the hydrogen ion than for nitrite and nitrate. These results (removal efficiency and final effluent concentration of NH_4_^+^–N) are consistent with the removal efficiency and final effluent concentration of NH_4_^+^–N reported by Ruiz-Martinez et al. (2012) for a culture of *Scenedesmus* sp. collected from freshwater bodies in a synthetic medium (removal rates of 13.5–4.2 mg/L*day). Studies also show NH_4_^+^–N removal efficiency of 85.63% (da Fontoura et al. 2017), 92–99% (Katırcıoğlu Sınmaz, Erden, and Şengil 2022; Gao et al. 2015; P. Praveen and Loh 2015; Su et al. 2016), and NH_4_^+^-N removal efficiency of 100% (Shayesteh et al. 2021; W. Zhou et al. 2012) by *Chlorella* sp. used for secondary wastewater treatment. Furthermore, almost complete NH_4_^+^–N removal efficiency was also achieved by *Scenedesmus* species (F. Ji et al. 2014). *Scenedesmus* species microalgae grown using effluent from partially treated breweries by the USAB system showed a progressive increase in removal efficiencies and reached 99% at the end of the experiment. It was also reported that microalgae usually prefer NH_4_^+^–N as a main source of inorganic nitrogen (Cai et al. 2013; Yirgu et al. 2020; S. Zhou et al. 2014).

#### Phosphorous and orthophosphate removal efficiency and uptake by microalgae

A high phosphorus or phosphate concentration that originates mainly from stomach contents causes eutrophication of the receiving water bodies when released without proper treatment (Abideen A. et al. 2020). The 3^rd^ essential macronutrient microalgae require for their growth next to carbon and nitrogen is phosphorus, which nearly accounts for 0.5–4% of their biomass (Subramaniyam et al. 2016). The microalgae assimilate the TP in the form of PO_4_^−3^–P through the energy consumption process (Rasoul-Amini et al. 2014; Chaudhary et al. 2018).

**Fig. 5:**
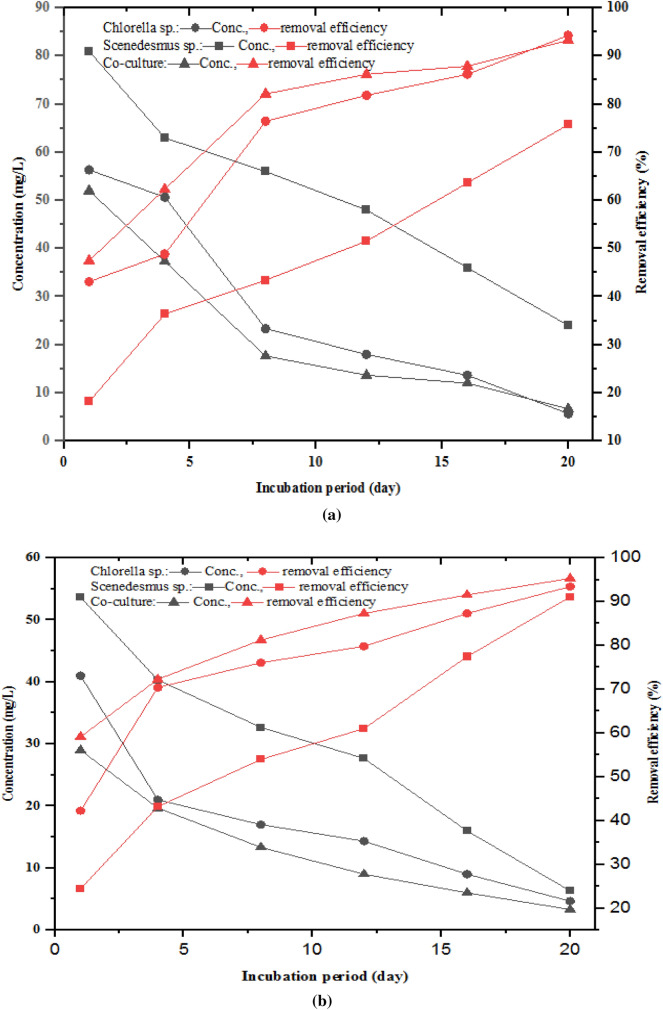
Trends of total phosphorous (**a**) and ortho-phosphate (**b**) concentration and removal efficiency during the incubation period

**Fig. 6:**
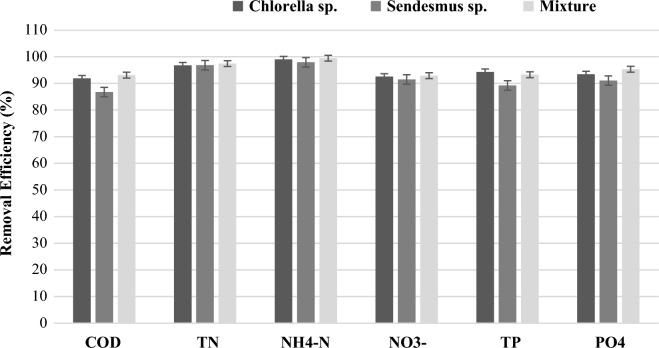
Nutrient and organic matter removal efficiency by microalgae photobioreactor

The variation of TP and PO_4_^−3^-P concentrations and RE during the experimental period is shown in Fig. 5 a and b, respectively. The concentrations of TP decreased with time, while the RE increased for all the treatments. On the 4^th^ day of the experimental period, about 50, 36, and 62% removal efficiency of TP was achieved for the two-phase AD effluent treated with *Chlorella*, *Scenedesmus*, and co-culture, respectively. The final effluent TP concentration and RE by *Chlorella*, *Scenedesmus*, and co-culture were found to be 5.67 ± 0.58, 10.67 ± 3.61, 6.67 ± 2.52 mg/L, and 94.28%, 89.22%, and 93.27%, respectively. The final microalgae photobioreactor effluent PO_4_^−3^–P concentrations were 4.67 ± 1.53, 6.33 ± 3.21, and 3.33 ± 1.53 mg/L, with a removal efficiency of 91.08, 93.43, and 95.31% for *Chlorella*, *Scenedesmus*, and their mixture, respectively (Fig. 6). The removal rate for both TP and PO_4_^−3^–P was high in the 1^st^ eight days and gradually decreased, as revealed in Fig. 6, which is attributed to the uptake of the TP and PO_4_^−3^–P by microalgae for biomass formation.

Furthermore, AD effluent phosphorous level reduction by microalgae is mainly due to the phosphates storage in cytoplasmic presence in the form of polysaccharides, polymers, fatty materials, reproducing biomass, biosorption to the cell wall (Valchev and Ribarova 2022), and luxury reserves as polyphosphate in suitable circumstances. Similarly, other previous studies also indicated the phosphorous removal by microalgae was through assimilation in growing and duplicating biomass, luxury reserve in aerobic conditions as a source of energy for anaerobic conditions, and biomass used as a bioenergy source (Cormier 2010; Solovchenko et al. 2016; Rybicki 1997). Studies have shown different TP removal efficiency ranges between 70 and 90% (Radin, Saphira, and Mohamed 2017; P. Praveen and Loh 2015; D. Hu et al. 2021) and 85 and 96.43% (Katırcıoğlu Sınmaz et al. 2022; Gao et al. 2015; Zheng et al. 2019; Kim et al. 2013) for PO_4_^−3^–P by *Chlorella vulgaris*. Lower (31 to 70%) TP removal efficiency by *Scenedesmus* species (AlMomani and Örmeci 2016; Yirgu et al. 2020) and 71.29% of PO_4_^−3^–P (Usha et al. 2016), while higher TP RE of 89–97% (S. Zhu et al. 2022; da Fontoura et al. 2017; W. Zhou et al. 2012; Shayesteh et al. 2021; Ajala and Alexander 2020) and 100% (M. K. Ji et al. 2013) were reported. Likewise, lower PO_4_^−3^–P removal efficiencies between 12–21%, 22–83%, and 57–85% (D. Hu et al. 2021) by *Chlorella* species, *Scenedesmus* species, and the co-culture of the two, respectively, while TP removal efficiencies of *Chlorella* species were 62.5–74.7%, 75% by *Scenedesmus*, and 86% by the co-culture (Asmare, Demessie, and Murthy 2014) were also previously indicated. TP removal efficiency of 100% by *Chlorella* and *Scenedesmus* species noted in M. K. Ji et al. (2013), is in line with the findings of this study. Another study by Qin et al. (2016) reported that microalgae co-cultures or consortia showed a better removal efficiency (91–96%) than monocultures of *Chlorella* species (87%). Similarly, in agro-industry processing effluent treated by the two microalgae sp., phosphorus removal efficiency of 20–100% was reported (Cai et al. 2013). Rasala and Mayfield (2015) noted that phosphorus uptake by microalgae from AD effluent is stored in the form of polyphosphate in the microalgae cell. Slaughterhouse wastewater treated by *Chlorella and Scenedesmus* showed a removal efficiency of 69% of PO_4_^−3^–P (Y. Hu et al. 2019a, b). Regarding the removal rates of TP and PO_4_^−3^–P, 0.326 and 0.290 mg/L*day were reported by microalgae used for treating anaerobic digester effluent, respectively. The findings of the study revealed that the TP concentrations attained at the end of the experimental or cultivation period fulfilled the permissible discharge limit for wastewater treatment plant effluent standard suggested by EEPA.

#### Organic matter removal efficiency

**Fig. 7:**
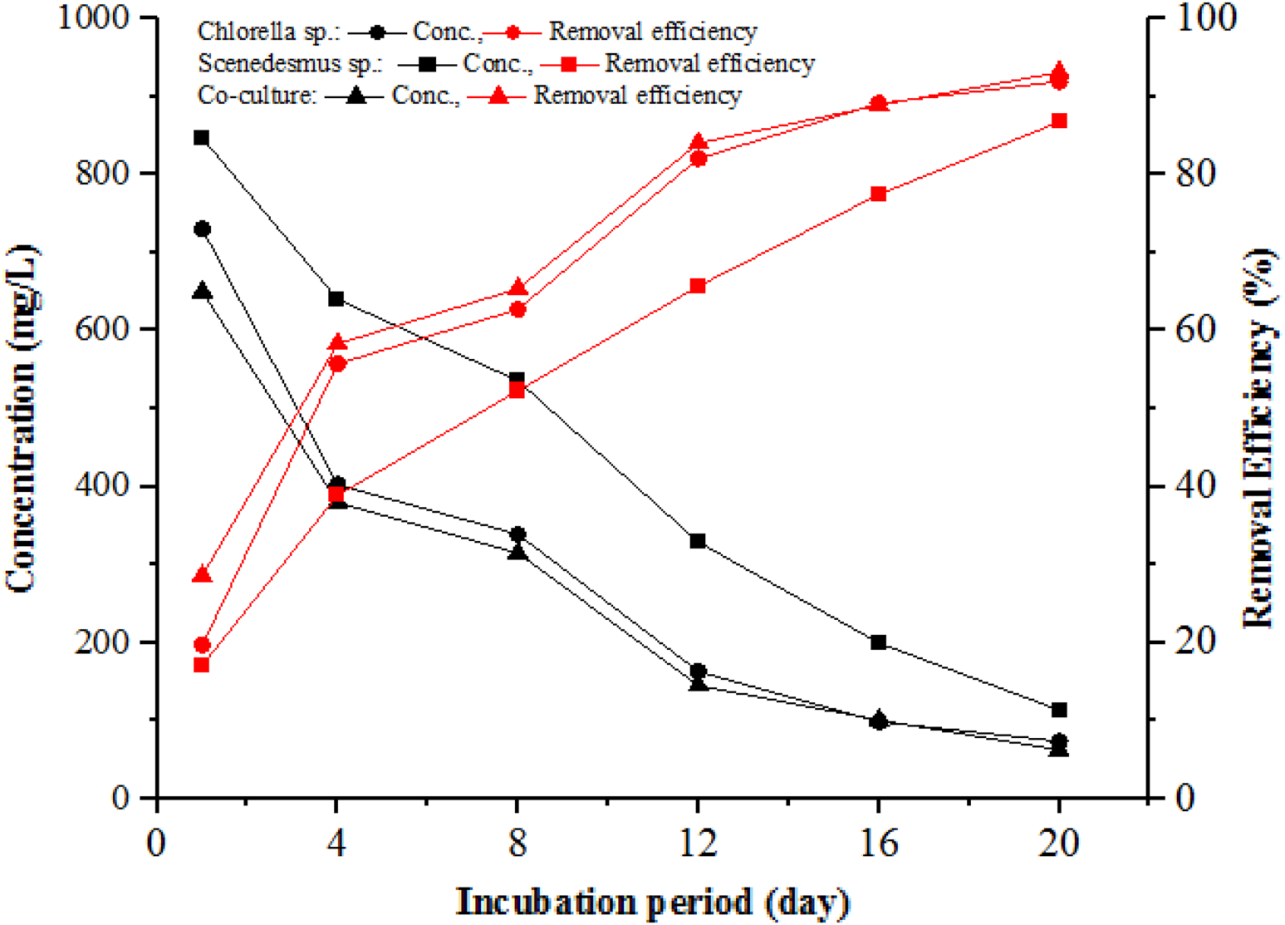
COD concentration and removal efficiency variation during the incubation period

Microalgae such as *Chlorella and Scenedesmus* microalgae, as well as the co-culture, can grow on organic carbon sources via assimilation (Şirin and Sillanpää 2015). The COD level treated by microalgae photobioreactors is another essential parameter, as it is indicative of the strength of the effluent and the quantity of oxygen that can be consumed for its oxidation (Otondo et al. 2018; Nagarajan et al. 2019; Choi and Lee 2012). Accordingly, the COD concentration in the two-phase AD effluent was used as a carbon source for the microalgae’s growth. Effluent COD concentration variation during the experimental period in which *Chlorella*, *Scenedesmus*, and co-culture microalgae were used to treat the two-phase AD effluent is shown in Fig. 7. COD removal efficiency of more than 50 and 80% was achieved after the 4th and 8th days of the incubation or experimental period in all treatments. The COD level in all treatments showed a continuous decrease in the first 15 days of treatment. The final photobioreactor effluent COD concentrations were 73.67 ± 6.51, 120.67 ± 6.51, and 62.67 ± 4.73 mg/L, with removal efficiencies of 87, 92, and 93% for *Chlorella*, *Scenedesmus*, and co-culture, respectively. The variation of mean COD concentrations in the treatments was significant between *Chlorella* and *Scenedesmus* (*p*-value = 0.01), *Chlorella* and the co-culture (*p*-value = 0.02), but not significant (*p*-value = 0.05) between *Scenedesmus* and the co-culture. The final concentrations of COD are below the wastewater treatment plant or slaughterhouse effluent discharge limit of Ethiopia (250 mg/L) in all treatments. The decrease in COD concentrations with incubation period is attributed to the uptake and usage of COD by microalgae as a source of organic carbon for their cell growth and biomass production, in addition to CO_2_. The COD uptake for *Chlorella*, *Scenedesmus*, and co-culture was 41.82, 39.47, and 42.37 mg/L*day, respectively (Fig. 7). In general, the concentration of COD in two-phase AD effluent is typically reduced during the incubation of microalgae.

Previous studies also showed that microalgae use the organic matter (COD) in partially treated (AD effluent) agro-processing industry effluent for their cell growth (Ding et al. 2015; B. Wang et al. 2012). The COD removal efficiency values of 87–93% noted in this study were consistent with the other study findings using *Chlorella* species and *Scenedesmus* species in wastewater treatment systems, indicating the microalgae and their co-culture used in this study were capable of effectively growing in partially treated slaughterhouse wastewater (Hernández et al. 2016; Choi and Lee 2012; Otondo et al. 2018). A COD removal efficiency of 89–99% using *Chlorella* sp. was reported in previous studies by S. Zhu et al. (2022); Zheng et al. (2019); Mehta and Chakraborty (2022), which is comparable to this study finding. A study conducted using *Scenedesmus* sp. for treating agro-processing industry wastewater reported COD and BOD removal efficiency of 75–80% and 82%, respectively (Usha et al. 2016; da Fontoura et al. 2017). COD removal efficiency of 64.9–76% (Abdel-Raouf et al. 2012; AlMomani and Örmeci 2016; Yang et al. 2015; Liu et al. 2022) and 80–85% (Cai et al. 2013; D. Hu et al. 2021) by *Chlorella* species from agro-processing industry wastewater, which is lower than this study result. The difference may be due to the reactor type and organic matter sources for microalgae-based wastewater treatment, which is slaughterhouse wastewater effluent treated by two-phase in this study and municipal wastewater in their study. Another study by Qin et al. (2016) reported that microalgae co-cultues or consortiums showed better COD removal efficiency (91–96%) than monocultures of *Chlorella* species, and Hena et al. (2015) reported better growth and stability by conglomerates of microalgae than single strains with 98% nutrients and COD removal efficiency from dairy wastewater.

### Microalgae biomass production

**Fig. 8:**
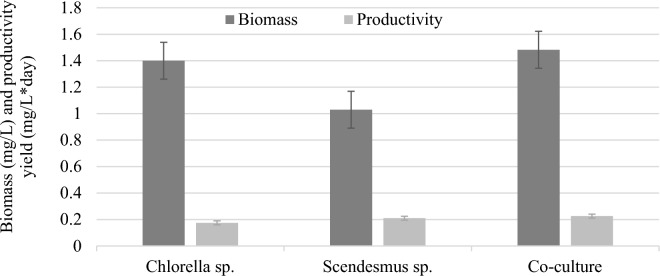
Biomass production and productivity of microalgae

The average microalgae biomass produced as well as productivity by each treatment are indicated in Fig. 8. As illustrated in Fig. 8, there was noticeable growth of *Chlorella*, *Scenedesmus*, and co-cultures in two-phase AD effluent. *Chlorella, Scenedesmus, and* co-culture biomass concentrations at the start (end) of the experiment were 0.11 (1.4 ± 0.1), 0.08 (1.17 ± 0.12), and 0.09 (1.5 ± 0.13) g/L, respectively. The minimum and maximum biomass productivity of *Chlorella*, *Scenedesmus*, and co-culture were 0.095 and 0.26, 0.26 and 0.34, 0.34 and 0.50 g/L*day, respectively. The average biomass yields were significantly higher for the co-culture compared to the individual microalgae (*p* ≤ 0.05). The final biomass attained for this study may be attributed to the high consumption of the dissolved inorganic and organic N and P in the two-phase AD effluent that are available in the form of PO_4_^−3^–P, NH_4_^+^–N, and NO_3_^−^–N, as well as TN and TP.

Associating this study’s results with the works of other researchers, the following were distinguished: For example, the microalgae’s biomass obtained in this study is lower than that reported by Elvira Ziganshina and Svetlana Bulynina (2022) for both *Chlorella* species (2.13–3.26 g/L) and *Scenedesmus* sp. (1.46–2.33 g/L). In the research work by Fernandes et al. (2022), 0.49 g/L and 0.23 g/L of *Chlorella* *vulgaris* and *Scenedesmus* biomass production, respectively, were reached in a digestate of pig manure cultivated in flasks, but a maximum ultimate concentration of 2.49 g/L biomass was reported by Kisielewska and Bordiean (2022) for *Chlorella vulgaris* cultivated in centrifuged agricultural digestate (media-based type) in tubular photobioreactors. Other scholars have shown that the highest microalgae biomass of up to 8.08 g/L of *Chlorella sorokiniana* can be found when cultivated in 50% diluted swine wastewater using glass-made vessels (photobioreactors) (Chen et al. 2020). A study by Hilares et al. (2021) that was conducted in batch mode for the cultivation of *Chlorella vulgaris* in acid-precipitated poultry slaughterhouse wastewater reported comparable biomass production of 1.2 g/L via 83% COD removal efficiency. Microalgae biomass productivity reported for microalgae grown on anaerobically treated agro-processing industry effluent varies between 0.1–0.6, 0.2–0.8, and 0.3–1.0 g/L for *Chlorella* sp., *Scenedesmus* sp., and their co-culture, respectively (Ziganshina, E.E.; Bulynina, S.S.; Yureva, K.A.; Ziganshin 2022), which is comparable to this study results. To this end, researchers also noted that *Chlorella* and *Scenedesmus* species can grow competently in anaerobically digested agro-processing industry effluent (Bohutskyi et al. 2015; Zuliani et al. 2016; Kobayashi et al. 2013). On the other hand, *Chlorella vulgaris, Scenedesmus obliquus,* as well as *Spirulina platensis* have been reported to possess the highest growth rates and nutrient removal rates, among other microalgae, when cultured in anaerobically digested swine wastewater (Ayre et al. 2017; L. Wang et al. 2010; Xu et al. 2015; Kuo et al. 2015). Nevertheless, the variation in the attained microalgae biomass yields and productivity with the result of other studies or works would be due to the difference in parameters such as CO_2_ supply, light intensity, temperature, type of bioreactor, experiment duration, origin, as well as anaerobic digester effluent characteristics during the experiment, which can directly affect the microalgae growth.

From the results, it is evident that the pollutant removal efficiencies of *Chlorella*, *Scenedesmus* species, and co-culture varied significantly (*p* ≤ 0.05) (co-culture is higher). Similarly, co-culture showed higher microalgae biomass production. Hence, co-culture can be used if the initiative of producing microalgae biomass is exclusively to extract crude lipids from microalgae biomass for biodiesel production, compared to monoculture of *Chlorella* species and *Scenedesmus* species. Likewise, in the two-phase effluent treatment process investigated in this study, *Chlorella* species and co-culture can be optional. Furthermore, the co-culture of *Chlorella* and Scenedesmus species has achieved higher removal efficiency compared to the monocultures, regardless of the factors (Table 1 and Fig. 6). The higher pollutant removal efficiency and biomass yield of co-culture were mainly due to the cooperative and competitive interaction of the individual microalgae’s as well as their resistance to predators. In this regard, many authors findings showed that *Chlorella* and *Scenedesmus* are the most effective species for pollutant removal purposes (Asmare et al. 2014; Hameed 2007), though co-culture outlay in both pollutant removal and microalgae biomass production.

## Conclusion

The microalgae species *Chlorella* and *Scenedesmus* collected from the local lake, as well as the co-culture used to treat slaughterhouse effluent treated in two-phase AD, have shown an encouraging removal efficiency of the nutrients and organic matter. Removal efficiencies between 86.74–93.11%, 96.74–97.47%, 91.49–92.91%, 97.94–99.46%, 89.22–94.28%, and 91.08–95.31% were achieved for COD, TN, NO_3_^−^–N, NH_4_^+^–N, TP, and PO_4_^−3^–P, by *Chlorella* species, *Scenedesmus* species, and their co-culture, respectively. Congruently, *Chlorella* species, *Scenedesmus* species, and co-culture biomass yield and productivity were 1.4 ± 0.1, 1.17 ± 0.12, 1.5 ± 0.13 g/L, and 0.18, 0.21, and 0.23 g/L*day, respectively. Henceforth, two-phase AD effluent supported microalgae biomass production through residual organic matter and nutrient removal to the required level. For all the parameters, the photobioreactor effluent concentration was below the slaughterhouse industry discharge limit of the country (Ethiopia). It can be concluded that the microalgae co-culture produces higher microalgae biomass and productivity via substantial removal efficiencies of pollutants than the monocultures. Furthermore, integration of microalgae photobioreactors into AD systems as a polishing step demonstrates sustainable agro-processing industry wastewater treatment and biomass production as well as an exercise of circular bioeconomy.

## Data Availability

All the data and materials used in this manuscript are included in this document.

## Abbreviations

AD: Anaerobic digestion
ANOVA: Analysis of variance
APHA: American Public Health Association
BBM: Bold’s Basal Medium
BOD: Biological oxygen demand
COD: Chemical oxygen demand
EC: Electrical conductivity
HRT: Hydraulic retention time
NH_4_-N: Nitrogen ammonium
OD: Optical density
OLR: Organic loading rate
ORP: Oxidation reduction potential
RE: Removal efficiency
Rr: Removal rate
SCOD: Soluble chemical oxygen demand
SEM: Scanning electron microscope
SHWW: Slaughterhouse wastewater
TCOD: Total chemical oxygen demand
TDS: Total dissolved solid
TN: Total nitrogen
TSS: Total suspended solids
TN: Total phosphorous
TVFA: Total volatile fatty acids
USAB: Upflow sludgebed
VFA: Volatile fatty acids
